# Differences in GPR30 Regulation by Chlorotriazine Herbicides in Human Breast Cells

**DOI:** 10.1155/2016/2984081

**Published:** 2016-02-03

**Authors:** Colin P. Florian, Shelly R. Mansfield, Jennifer R. Schroeder

**Affiliations:** Department of Biology, Millikin University, Decatur, IL 62522, USA

## Abstract

Over 200,000 cases of invasive breast cancer are diagnosed annually; herbicide contaminants in local water sources may contribute to the growth of these cancers. GPR30, a G protein coupled receptor, was identified as a potential orphan receptor that may interact with triazine herbicides such as atrazine, one of the most commonly utilized chlorotriazines in agricultural practices in the United States. Our goal was to identify whether chlorotriazines affected the expression of GPR30. Two breast cancer cell lines, MDA-MB-231 and MCF-7, as well as one normal breast cell line, MCF-10A, were treated with a 100-fold range of atrazine, cyanazine, or simazine, with levels flanking the EPA safe level for each compound. Using real-time PCR, we assessed changes in GPR30 mRNA compared to a GAPDH control. Our results indicate that GPR30 expression increased in breast cancer cells at levels lower than the US EPA drinking water contamination limit. During this treatment, the viability of cells was unaltered. In contrast, treatment with chlorotriazines reduced the expression of GPR30 in noncancerous MCF-10A cells. Thus, our results indicate that cell milieu and potential to metastasize may play a role in the extent of GPR30 response to pesticide exposure.

## 1. Introduction

Over 1.1 billion pounds of pesticides is used annually in the USA, accounting for twenty-five percent of the total world usage [1]. These environmental chemicals may have dire consequences upon tissue development if brought in contact with several hormone-dependent tissues such as breast and ovarian, and this draws great concern as one in every eight American women is diagnosed with some form of breast cancer [2]. Triazine exposure has been highly correlated with the incidence of ovarian cancer [3]. Atrazine has also been known to cause mammary tumors in rats [4]. Cyanazine is considered moderately toxic showing decreased cell viability and maternal body weight and can cause depression [5]. Simazine is considered slightly nontoxic, although it is a reported mutagen in human lung cells, causes tumors in mammary and thyroid tissues in rats, and decreases birth weight while increasing fetotoxicity in rabbits [6, 7].

Through RT-PCR analysis, Albanito et al. [3] showed that estrogen-responsive genes that ultimately cause proliferation were upregulated in cancerous ovarian cell lines upon exposure to atrazine. More importantly, they showed that such genes were upregulated in cancerous breast cells that lack classical estrogen receptors ER*α* and ER*β* [8], indicating a potential orphan receptor able to bind to atrazine and elicit intracellular changes. GPR30, a membrane-bound G protein coupled receptor, has been recognized as both a nonclassical receptor for estrogen and a potential receptor for triazines [9]; it is able to induce gene transcription through a variety of cellular pathways including increasing calcium and cAMP [10].

A study of GPR30 presence in MCF-7 cells shows receptor localization in the endoplasmic reticulum through fluorescently labelled estrogen derivatives [11]. Filardo et al. showed that the cancerous breast cell line MDA-MB-231 expressed low levels of GPR30 [12, 13]. Upregulation of estrogen dependent genes has been observed in these cells through the induction of MAP kinase pathways through GPR30 binding [14]. Cronan et al. [15] found that six of seven MAP3-kinases they tested regulated tumor growth and metastasis in MDA-MB-231 cells, suggestive of GPR30 presence [10]. Recent studies have indicated that although MCF-10A cells may lack or have very low levels of classical ER, they do express and regulate gene expression through GPR30 [16, 17].

Rather than focusing upon estrogen-based activation of GPR30, in this study we have examined the potential to alter GPR30 gene expression through exposure to triazine class herbicides in two cancerous breast cell lines and one normal breast cell line. MCF-10A noncancerous and MCF-7 and MDA-MB-231 cancerous cell lines were treated with three different concentrations of atrazine, cyanazine, and simazine, flanking the levels considered safe in drinking water by the United States Environmental Protection Agency (EPA). Our hypothesis is that GPR30 expression will increase as the herbicides act as ligands for the receptor, thus upregulating its own expression. However, we expect to see discrete differences in the cell lines and expect that these differences may allow for clear separation between noncancerous cells and those with the ability to metastasize.

## 2. Materials and Methods

### 2.1. Cell Culture and Maintenance

All cell lines were obtained from the lab of Dr. Ann Nardulli (University of Illinois at Urbana-Champaign, Urbana, IL), subcultured from stocks originally obtained from ATCC (Manassas, VA). MDA-MB-231 human epithelial adenocarcinoma cells were maintained in DMEM/F-12 media supplemented with 10% fetal calf serum and antibiotics (50 IU/mL penicillin, 50 *μ*g/mL streptomycin, and 5 *μ*g/mL gentamycin sulfate) at 37°C in a humidified, 5% CO_2_ environment. MCF-7 human epithelial adenocarcinoma cells were maintained in Modified Eagle's Media supplemented with 5% calf serum and antibiotics, while assays were performed in either maintenance media or reduced medium containing phenol red-free Modified Eagle's Media and supplemented with 5% charcoal dextran-stripped calf serum and antibiotics. MCF-10A cells were maintained in DMEM/F-12 media supplemented with 5% horse serum, MEGS supplement, and 0.1 *μ*g/mL cholera toxin.

### 2.2. Cell Viability

MCF-7 and MDA-MB-231 cells were seeded into 96-well plates either in maintenance media (MCF-7 and MDA-MB-231) or in reduced media (MCF-7). Twenty-four hours after plating, the media were replaced with maintenance (MCF-7 and MDA-MB-231) or reduced (MCF-7) media containing atrazine, simazine, or cyanazine at 0.1-, 1-, and 10-fold the EPA safe levels (Table 1) or DMSO control. Atrazine was added at 0.3, 3.0, or 30 ppb (*μ*g/L; 0.64–64 *μ*M), cyanazine was added at 0.1, 1.0, or 10 ppb (0.17–17 *μ*M), and simazine was added at 0.4, 4.0, or 40 ppb (0.92–92 *μ*M). After twenty-four hours, 3 *μ*g resazurin was added and allowed to incubate for three hours prior to reading absorbance at 570 and 595 nm on iMark Microplate Absorbance Reader (Bio-Rad, Hercules, CA). We performed univariate analysis of variance (ANOVA) using SPSS (IBM SPSS Statistics, IBM Corp., Armonk, NY).

### 2.3. GPR30 Expression Analysis

Prior to treatment, cells were seeded into 12-well plates in their respective maintenance media. Twenty-four hours after plating, the media were replaced with maintenance media containing atrazine, simazine, or cyanazine at 0.1-, 1-, and 10-fold the EPA safe levels (Table 1), or DMSO control. After twenty-four hours, RNA was harvested using RNAzol (Molecular Research Center, Inc., Cincinnati, OH) per manufacturer's recommendations. Extracted nucleotides were treated with DNase, and cDNA was synthesized using RT-PCR with random primers using GoScript Reverse Transcriptase (Promega, Madison, WI) according to manufacturer's recommendations.

Real-time PCR was run in triplicate for 40 cycles using a Bio-Rad iCycler iQ (Bio-Rad, Hercules, CA). GAPDH was used as a reference gene. GAPDH (ReadyMade Primer Sequence, IDT DNA Technologies, Coralville, IA) and GPR30 primers [19] for real-time PCR were obtained from IDT DNA Technologies (Coralville, IA) with the following sequences:

 GAPDH forward: 5′-ACCACAGTCCATGCCATCAC-3′,
 GAPDH reverse: 5′-TCCACCACCCTGTTGCTGTA-3′,
 GPR30 forward: 5′-AGTCGGATGTGAGGTTCAG-3′,
 GPR30 reverse: 5′-TCTGTGTGAGGAGTGCAAG-3′.

The ΔCt value was calculated by subtracting the control gene amplification cycle from the GPR30 amplification cycle within each treatment. To calculate ΔΔCt, ΔCt value for the control treatment (DMSO) was subtracted from each experimental treatment. Fold change was calculated using 2ΔΔCt [20]. We performed univariate analysis of variance (ANOVA) using SPSS (IBM SPSS Statistics, IBM Corp., Armonk, NY).

## 3. Results

To ensure that treatment with pesticides did not cause dramatic changes in cell viability, we treated MCF-7 breast cancer cells with three concentrations of atrazine, cyanazine, or simazine. Concentrations selected flanked the maximum contamination levels for drinking water set by the US EPA (Table 1).

**Figure 1:**
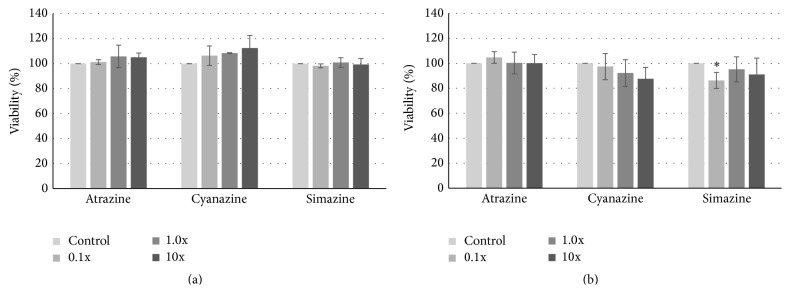
Cell viability of MCF-7 cells treated with triazine herbicides. MCF-7 human breast cancer cells were treated with 0.1- to 10-fold concentrations of the EPA safe levels of atrazine, cyanazine, or simazine for 24 hours in reduced (a) or maintenance (b) media. Cell viability was determined by levels of reduction of resazurin to resorufin by metabolically active cells. Percent viability of cells ± standard deviation is shown, with the DMSO-treated controls normalized to 100 percent. Statistically significant changes in viability compared to the vehicle control are indicated (^*∗*^ *p* < 0.05).

Cell viability was measured using resazurin, which is reduced to resorufin in the presence of metabolically active cells [21]. We maintained MCF-7 cells in a reduced medium to eliminate hormonal effects and treated them with atrazine, cyanazine, or simazine at 0.1-, 1-, or 10-fold the EPA safe level in drinking water for twenty-four hours. We observed no statistically different changes in cell viability in the presence of atrazine or cyanazine and only a small difference when comparing 0.1x simazine to the DMSO control (Figure 1(a)). As minimal media can induce cellular stress, we repeated the viability assay using MEM with calf serum. As with the reduced medium, we noted no statistical difference in viability at any concentration of any of the three triazines (Figure 1(b)). Thus, we proceeded with the richer maintenance medium for the remainder of our study.

To assess the ability of chlorotriazines to induce changes in GPR30 mRNA expression, we then treated MCF-7 cells with the same three concentrations of atrazine, cyanazine, and simazine. mRNA was harvested and utilized to synthesize cDNA, which was then selectively amplified using primers specific to GPR30 using previously published primers by Girgert et al. [19] or a genomic control, GAPDH. As a housekeeping gene, GAPDH levels should not fluctuate upon treatment with the pesticides.

**Figure 2:**
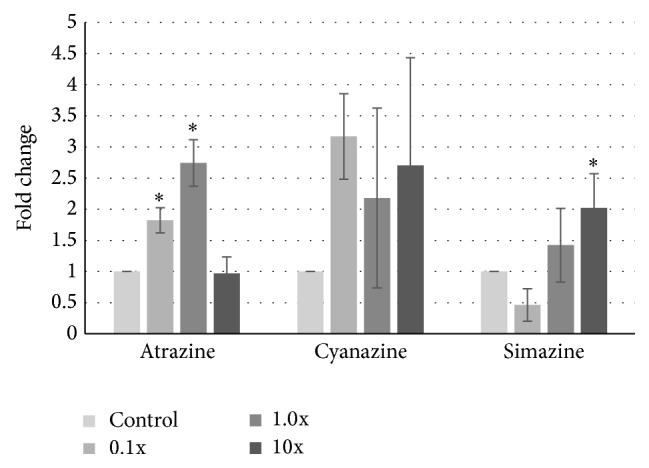
Increases in GPR30 expression in MCF-7 cells after atrazine exposure. MCF-7 human breast cancer cells were treated with 0.1- to 10-fold concentrations of the EPA safe levels of atrazine, cyanazine, or simazine for 24 hours. mRNA was harvested, cDNA was synthesized, and specific amplicons were prepared using primers to GAPDH (reference gene) or GPR30. Resulting fold changes in expression ± standard deviation are shown, with the vehicle control set at 1. Statistically significant changes in induction compared to the control are indicated (^*∗*^ *p* < 0.05).

Treatment with atrazine resulted in a change in the expression of GPR30. At the lowest concentration, we observed a modest 1.8-fold increase compared to treatment with the DMSO control, and at the EPA safe level the increase was 2.7-fold (Figure 2). However, at the highest concentration, levels fell to the untreated levels. Although we were unable to identify any statistically significant changes for cyanazine, a trend of increase was observed, with 2-3-fold increases in expression at all three concentrations. While not statistically significant, the 3.1-fold increase at 0.1x approached significance (*p* = 0.054). Treatment with simazine had no effect at low levels; however GPR30 expression was statistically higher at the 10x concentration (2.0-fold increase).

**Figure 3:**
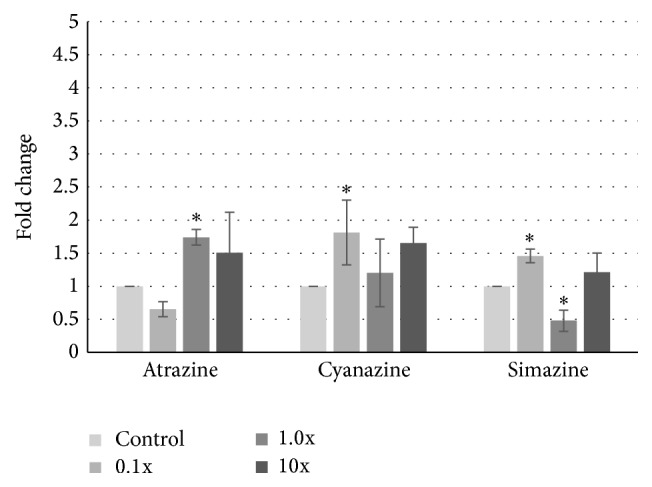
Change in GPR30 expression in MDA-MB-231 cells after atrazine exposure. MDA-MB-231 human breast cancer cells were treated with 0.1- to 10-fold concentrations of the EPA safe levels of atrazine, cyanazine, or simazine for 24 hours. mRNA was harvested, cDNA was synthesized, and specific amplicons were prepared using primers to GAPDH (reference gene) or GPR30. Resulting fold changes in expression ± standard deviation are shown, with the vehicle control set at 1. Statistically significant changes in induction compared to the control are indicated (^*∗*^ *p* < 0.05).

To identify whether these changes were a property of treating cancerous cells with triazines, we analysed GPR30 expression in another breast cancer cell line, MDA-MB-231, as well as the noncancerous MCF-10A cell line. As observed in Figure 3, treatment of MDA-MB-231 cells with pesticides also resulted in modest, 1.5- to 2-fold increases in GPR30 expression, although the patterns differed slightly from those observed in MCF-7 cells. We detected statistically significant increases in GPR30 expression at the EPA safe level of atrazine, but not at concentrations above or below that level. In contrast, increases were observed at the lowest concentration of cyanazine, and trends for increases were seen for both 1x and 10x concentrations, with the 10x approaching significance (*p* = 0.08). For simazine, increases were observed at the 0.1x concentration, while the 1x concentration resulted in statistically lowered levels of GPR30.

**Figure 4:**
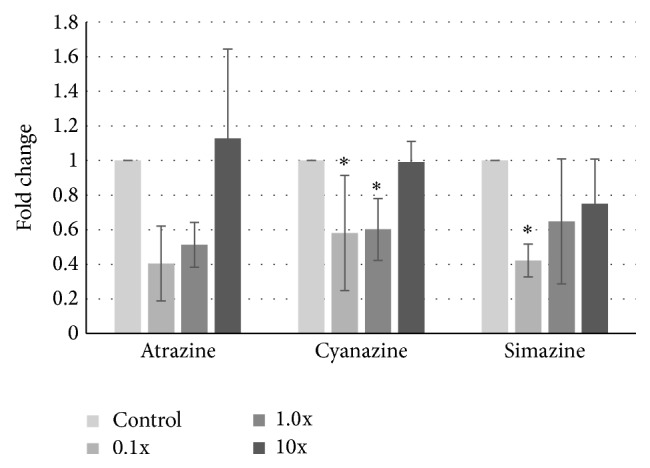
Change in GPR30 expression in MCF-10A cells after atrazine exposure. MCF-10A noncancerous breast cells were treated with 0.1- to 10-fold concentrations of the EPA safe levels of atrazine, cyanazine, or simazine for 24 hours. mRNA was harvested, cDNA was synthesized, and specific amplicons were prepared using primers to GAPDH (reference gene) or GPR30. Resulting fold changes in expression ± standard deviation are shown, with the vehicle control set at 1. Statistically significant changes in induction compared to the control are indicated (^*∗*^ *p* < 0.05).

We identified the most striking difference, however, in the noncancerous MCF-10A cells. In contrast to the cancerous cells, we detected significant decreases in GPR30 expression (Figure 4). For atrazine, a trend of decreased expression was seen, with levels below 0.5-fold expression which was nearly statistically significant (*p* = 0.064 and 0.084 for 0.1x and 1x concentrations, resp.). Cyanazine exposure also led to a reduction in GPR30 expression at both 0.1x and 1x concentrations. Simazine treatment significantly reduced GPR30 expression to 0.6-fold only at the lowest concentration, although the higher concentrations showed indication of reduced expression as well.

## 4. Discussion

GPR30 has been identified in a variety of tissues, many of which are ER*α*-positive, as reviewed in [10, 22]. There has been conflicting evidence regarding the ability of GPR30 to interact with 17*β*-estradiol; although there are several reports of altered gene expression through the interaction of estrogens to GPR30 [23, 24], others report a failure of atrazine to bind [25, 26]. Others report that much higher levels of estrogens and antiestrogens must be utilized to illicit a response [27]. Conversely, ER directly regulates GPR30 expression in MCF-7 cells [28, 29].

We were initially concerned that the rich medium used in our preliminary studies might influence gene expression, due to the reports of GPR30 being able to substitute for the estrogen receptor to drive gene expression [12, 28, 30], and that the phenol red in our medium might also trigger a response [31]. However, more recent studies have confirmed that the amount of phenol red in current maintenance media is not high enough to stimulate estrogen-responsive gene expression [32]. The lack of difference between treated cell growth in MCF-7 cells kept in a reduced medium (hormone-free and lacking phenol red) and our maintenance medium (containing serum and phenol red) indicates that the assays being performed are not dependent upon any estrogenicity of the triazines (Figure 1). This is in agreement with our previous work which examined the viability of MCF-7 and MDA-MB-231 cells after treatment with four herbicides at levels just above those deemed safe for daily consumption by the US EPA [18]. Our current study examines levels that overlap this work, yet it looks at a shorter treatment time (twenty-four instead of forty-eight hours). If atrazine, cyanazine, and simazine were to be working through estrogenic pathways in our study, we would have expected an increase in cell viability even at twenty-four hours of treatment, especially within cells carried in a reduced medium [33, 34]. In fact, recent work by de la Casa-Resino and Albanito has confirmed early studies by Connor et al. that chlorotriazine compounds do not bind to nor activate gene expression directly through the classical estrogen receptor [35–37]. This, combined with previous reports of altered growth and cellular responses when MCF-7 cells are in a deprived medium [38, 39], supports our going forward with our studies in a richer medium that promoted a more robust growth of cells.

Although the levels of triazine class herbicides used in this and our previous work did not alter cell growth, nor did it affect cellular migration [18, 40], we wanted to determine if genomic changes in GPR30 occurred with chlorotriazine exposure. In fact, upon examining the expression level of GPR30, the proposed receptor for these compounds, we did observe discrete differences in expression dependent upon cell milieu. While levels of GPR30 were reduced in the noncancerous MCF-10A cells when exposed to low levels of atrazine, cyanazine, and simazine (Figure 4), we saw striking differences in the cancerous cell lines. In both MDA-MB-231 and MCF-7 cells, we observed an increase, albeit modest, in GPR30 expression at these same concentrations (Figures 2 and 3). Interestingly, it has been proposed that one role of GPR30 is to antagonize growth of ER*α*-positive breast cancer cells, such as the MCF-7 cell line, yet support growth of ER-negative tumors [28, 41]. This may be through the ability of GPR30 to limit ER activity [42].

Despite the alteration in GPR30 expression upon triazine treatment, we would be remiss to associate these changes solely with the possible interaction of these pesticides with GPR30 alone. Albanito et al. have shown that atrazine is able to bind directly to GPR30 to activate Erk phosphorylation and gene expression [3, 36]. However, the altered expression of GPR30 in our study may or may not be due to direct interaction of these compounds with GPR30 itself. GPR30 can be upregulated through several ligands such as EGF, IGF-1, and VEGF [29, 43, 44] and may be activated by progesterone as well [45, 46]. GPR30 has also been shown to activate the c-Fos/AP1 pathway, and these trans-acting factors in turn upregulate GPR30 [30, 47–49].

We believe that it is possible that the differences we observe in GPR30 regulation by triazines may be indicative of a cell's metastatic state. The ability of atrazine in particular to cause a change in gene regulation is also consistent with the previously observed ability of xenoestrogens, especially chlorotriazines [24, 50], although the roles of cyanazine and simazine have been less well studied than atrazine. Our previous studies did not indicate changes in growth rates when we utilized atrazine, cyanazine, and simazine at levels that far exceeded the EPA safe level in drinking water [18]. Likewise, when assessing the ability to respond after artificial wound induction, MCF-7 cells showed no change from the DMSO control when exposed to triazines, with less than 40% regrowth over 72 hours [40]; this was in contrast to the effects of estradiol which stimulated regrowth and migration. MDA-MB-231 cells exhibited initial regrowth at a rate below the control, while MCF-10A cells were fully regrown in that same timespan regardless of treatment used (hormonal or pesticide). This data correlates well with examination of primary tumors, where GPR30 overexpression is observed in high-grade and aggressive breast and ovarian cancers [27, 51–53]. Activation of GPR30 triggers an intracellular signalling cascade, leading to increased levels of cAMP and activated protein kinase A [23, 24, 54], as well as activation of Erk [8]. In several studies, it has been shown that the activation of Erk leads to a transfer from the G1 to S phase, thus leading to cell division [55, 56]. These pathway activations may allow for the progression of breast cancer [57–59], and this has been directly suggested by Vivacqua et al. [44] with overexpression of GPR30 in the development of aggressive phenotypes in estrogen-dependent breast cancers.

Taking those results in context with our current study, we see that a downregulation of GPR30 after triazine exposure in MCF-10A may be protective, correlating with normal growth patterns and an inability to respond to further pesticide exposure through the cAMP pathway. This is a pathway often utilized by steroid hormones, where the compounds downregulate their own receptors [60–63] to limit cellular responses. However, metastatic cells, through upregulation of GPR30, see a shift in the ability of the cells to regrow. We may expect that the upregulation of GPR30 allows for the cAMP-induced “brake” to be placed on the Erk pathway, thus forcing cells to rely upon induction of MAP kinase pathways for further growth [14]. Thus, while there are conflicting reports as to the toxicity of chlorotriazine herbicides [4], the potential for damage may depend upon the existing cell milieu.

## 5. Conclusion

Treatment with triazines did not alter cell viability in MCF-7 cells; however, we observed upregulation of GPR30 mRNA. This pattern of upregulation was also seen in the cancerous MDA-MB-231 cells but not in the noncancerous breast cell line MCF-10A. Thus, the metastatic potential of a cell line may play a role in the ability of cells to regulate the amount of GPR30 transcript produced when cells are exposed to an environmental ligand.

## Figures and Tables

**Table 1:** Pesticide use levels based upon US EPA maximum contamination levels (MCL) for drinking water. Modified from Rich et al. [18].

	MCL (*µ*g/L)	0.1x (*µ*g/L)	1x (*µ*g/L)	10x (*µ*g/L)
Atrazine	3	0.3	3	30
Cyanazine	1	0.1	1	10
Simazine	4	0.4	4	40
